# Triple blockade of EGFR, MEK and PD-L1 has antitumor activity in colorectal cancer models with constitutive activation of MAPK signaling and PD-L1 overexpression

**DOI:** 10.1186/s13046-019-1497-0

**Published:** 2019-12-16

**Authors:** S. Napolitano, N. Matrone, A. L. Muddassir, G. Martini, A. Sorokin, V. De Falco, E. F. Giunta, D. Ciardiello, E. Martinelli, V. Belli, M. Furia, S. Kopetz, F. Morgillo, F. Ciardiello, T. Troiani

**Affiliations:** 1 Medical Oncology Department of Precision Medicine, University of Campania “Luigi Vanvitelli”, 80100 Naples, Italy; 2 Department of Gastrointestinal Medical Oncology, Division of Cancer Medicin0065, The University of Texas MD Anderson Cancer Center, 1515 Holcombe Blvd, Houston, TX 77030 USA; 3 Medical University of Vienna, Institute for Cancer Research, Borschkegasse 8A, 1090 Wien, Austria; 4 Vall D’Hebron Institute of Oncology (VHIO), Gastrointestinal and neuroendocrine tumor group, C/Natzaret 115-117, 08035 Barcelona, Spain; 5 Department of Biology, University of Naples Federico II, 80126 Naples, Italy

**Keywords:** Colorectal cancer, MEK inhibitor resistance, PD-L1 inhibitors

## Abstract

**Background:**

Molecular mechanisms driving acquired resistance to anti-EGFR therapies in metastatic colorectal cancer (mCRC) are complex but generally involve the activation of the downstream RAS-RAF-MEK-MAPK pathway. Nevertheless, even if inhibition of EGFR and MEK could be a strategy for overcoming anti-EGFR resistance, its use is limited by the development of MEK inhibitor (MEKi) resistance.

**Methods:**

We have generated in vitro and in vivo different CRC models in order to underline the mechanisms of MEKi resistance.

**Results:**

The three different in vitro MEKi resistant models, two generated by human CRC cells quadruple wild type for *KRAS*, *NRAS*, *BRAF*, *PI3KCA* genes (SW48-MR and LIM1215-MR) and one by human CRC cells harboring *KRAS* mutation (HCT116-MR) showed features related to the gene signature of colorectal cancer CMS4 with up-regulation of immune pathway as confirmed by microarray and western blot analysis. In particular, the MEKi phenotype was associated with the loss of epithelial features and acquisition of mesenchymal markers and morphology. The change in morphology was accompanied by up-regulation of PD-L1 expression and activation of EGFR and its downstream pathway, independently to *RAS* mutation status. To extend these in vitro findings, we have obtained mouse colon cancer MC38- and CT26-MEKi resistant syngeneic models (MC38-MR and CT26-MR). Combined treatment with MEKi, EGFR inhibitor (EGFRi) and PD-L1 inhibitor (PD-L1i) resulted in a marked inhibition of tumor growth in both models.

**Conclusions:**

These results suggest a strategy to potentially improve the efficacy of MEK inhibition by co-treatment with EGFR and PD-L1 inhibitors via modulation of host immune responses.

## Background

Colorectal cancer (CRC) is the second leading cause of cancer-related death worldwide [1]. In the past two decades, the introduction of novel targeted therapeutic agents and the better selection of patients have significantly changed treatment strategies for CRC and have brought to improved patient outcome [2, 3]. However, overall the progress has been more modest than had been hoped and metastatic colorectal cancer (mCRC) unfortunately remains for the majority of patients an incurable disease [4]. One of the major problems to more effective precision medicine-guided treatments in mCRC is the cancer genetic heterogeneity that is responsible of the selection and the expansion of cancer cell clones which are resistant to therapies [5, 6]. On the other hand, the high degree of genetic heterogeneity observed in CRC makes difficult to define clinical groups of patients that could be treated in a more effective personalized approach and to better define mechanisms of resistance to standard therapies [7–9].

Personalized treatments most often involve kinase inhibitors or monoclonal antibodies (moAbs) that target specific alterations known to drive the proliferation and survival of cancer cells [1]. In this scenario, the epidermal growth Factor receptor (EGFR) family plays a key role in tumor growth and progression by promoting a variety of functions including proliferation, survival, invasion, and immune evasion [2]. These therapies have improved patient responses, however, despite significant progress in strategies for cancer treatment, their use is limited by the presence of pre-existing intrinsic resistance mechanisms or by the ability of cancer cells to acquire resistance [3, 4, 6]. In this respect, the evolution of acquired resistance to anti-EGFR therapy can be defined as the consequence of a perturbation in a system in which most of the mutations that emerge upon treatment involve genes that are involved in the EGFR-driven signaling pathway [3]. In particular, we recently have demonstrated that the plethora of alterations that emerge at progression from treatment with the anti-EGFR moAb cetuximab, biochemically converge to reactivate the main EGFR downstream effector, the RAS-RAF-MEK-MAPK pathway [3, 10]. Based on this hypothesis, we have evaluated the ability of selective MEK inhibitors (MEKi) in overcoming cetuximab resistance in various human CRC models [10]. Combined treatment with cetuximab and MEKi induced synergistic anti-proliferative and pro-apoptotic effects coupled with MAPK and AKT inhibition and subsequently tumor growth inhibition and increase mice survival in in vivo human CRC models [10]. Moreover, in order to evaluate a possible strategy to prevent and/or delay the onset of resistance to anti-EGFR inhibitors we have investigated in three models of highly EGFR-dependent human colon cancer xenografts the effect of maintenance therapy with different kinases inhibitors that act downstream to the EGFR pathway after an induction cytotoxic treatment with cetuximab plus irinotecan [11]. In this respect, the combined treatment with cetuximab plus MEKi is able to prevent and/or overcome the resistance to anti-EGFR inhibitors [10, 11]. Therefore, MEK lies at a critical cross-road within the RAS/MAPK pathway and represents a convergence point of aberrant activation of different upstream signalling molecules [12]. Results from these studies have been promising and suggest that MEKi, whether alone or in combination with other anticancer therapies, may play a significant role in the management of this malignancy. However, some tumors after an initial benefit to MEKi started to re-growth underling the emergence of cancer cell resistance mechanisms [12]. Moreover, treatment MEKi as single agent failed to show any clinical benefit in mCRC patients [13]. For these reasons, to identify possible mechanisms of resistance we have generated and characterized different in vitro and in vivo MEKi-resistant colon cancer models.

In particular, in the present work, we have found an enhanced immune-reactive phenotype in MEKi resistant cells. These cells lost their epithelial features and gained more mesenchymal characteristics with concomitant overexpression of PD-L1 and of other immune-inflamed markers. This ‘inflamed’ immune profile was similar to CMS4 subtype of CRC [14–17]. Moreover, we also tried to better define the mechanisms underlying PDL1 regulation by finding a role for EGFR in PD-L1 expression modulation and subsequently in the development of acquired resistance to MEKi. To extend these in vitro findings, we performed an in vivo study using mouse MC38- and CT26-MEKi resistant syngeneic colon cancer models. In this respecrt. Combined treatment with MEKi, EGFR inhibitor (EGFRi) and PD-L1 inhibitor (PD-L1i) resulted in a marked inhibition of tumor growth in both cancer models.

Taken together, these results suggest a strategy to potentially improve the efficacy of MEK inhibition by co-treatment with EGFR and MEK inhibitors via modulation of host immune responses in MEKi-resistant models. These findings could define a potential role for immunotherapy-based strategies in a subgroup of human metastatic CRC.

## Methods

### Drugs

BAY86–9766 (a selective MEK 1/2 inhibitor) was kindly provided by Bayer Italy (Milan, Italy). For in vitro applications, BAY86–9766 was dissolved in sterile dimethylsulfoxide (DMSO) and the 10 mM stock solution was stored in aliquots at − 20 °C. Working concentrations were diluted in culture medium just before each experiment. For in vivo applications, BAY86–9766 was solubilized in 0.5% Tween-80 in sterile Phosphate Buffered Saline (PBS) before use. Erlotinib (EGFR inhibitor) was provided by Chemietek (Indianopolis, IN, USA) and for in vivo experiments was dissolved in 0.5% Methylcellulose before use. Mouse PD-L1 inhibitor (clone 10F.9G2) was provided by BioXcell (Lebanon, NH, USA) and for in vivo applications was solubilized in PBS before use.

### Cell lines

The human SW48 (catalogue number: HTL99020) and HCT116 (catalogue number: HTL99017) colon cancer cell lines were obtained from IRCCS “Azienda Ospedaliera Universitaria San Martino-IST Istituto Nazionale per la Ricerca sul Cancro” (Genova, Italy). The human LIM 1215 colon cancer cell line was obtained from Dr. Di Nicolantonio at Candiolo National Cancer Institute (Candiolo, Italy). The MC38 cell line derived from methylcholanthrene-induced C57BL6 murine colon adenocarcinoma and CT26 cell line derived from N-nitroso-N-methylurethane-induced BALB/c (H-2d) undifferentiated colon carcinoma were kindly provided from Dr. Kopetz at MD Anderson Cancer center (Houston, TX, USA).

SW48, LIM1215 and CT26 cells were grown in RPMI-1640 (Lonza) supplemented with 10% fetal bovine serum (FBS), 1 mM L-Glutamine, and Penicillin (100 μ/ml)–Streptomycin (0.1 mg/ml). HCT116 cell lines were grown in McCoy culture medium (Lonza, Cologne, Germany), supplemented with 10% FBS (Lonza), 1 mM L-Glutamine, and Penicillin (100 μ/ml)–Streptomycin (0.1 mg/ml). MC 38 cells were grown in DMEM (Lonza) supplemented with 10% FBS, 1 mM L-Glutamine, and Penicillin (100 μ/ml)–Streptomycin (0.1 mg/ml). All cell lines were grown in a humidified incubator with 5% of carbon dioxide (CO_2_) and 95% air at 37 °C. All cell lines were routinely screened for the presence of mycoplasma (Mycoplasma Detection Kit, Roche Diagnostics, Monza, Italy).

### Generation of MEKi resistant cell lines

Four- to six-week old female balb/c athymic (nu+/nu+) mice were purchased from Charles River Laboratories (Milan, Italy). The research protocol was approved and mice were maintained in accordance with the institutional guidelines of the University of Campania “Luigi Vanvitelli” Animal Care and Use Committee. Mice were injected subcutaneously (s.c.) with 3.5 × 10^6^ of SW48 cells that had been resuspended in 200 μl of matrigel (BD Biosciences, Milan, Italy). When tumors of approximately 200–300 mm^3^ in diameter were detected, mice were treated continuously by oral gavage with MEKi (BAY86–9766, 5 mg/kg, every day). Tumor size was evaluated twice per week by caliper measurements using the following formula: π/6 × larger diameter × (smaller diameter)^2^. When tumors were resuming growth despite MEKi treatment, mice were sacrificed and tumors were removed, were cut in several pieces and recovered in vitro by enzymatic treatment [18] to generate MEKi resistant cell lines (SW48-MR). Cells were passaged in culture at least three times before any further testing.

HCT116 and LIM1215 cells were treated continuously in vitro to increasing concentrations of MEKi (BAY86–9766) for a period of 6 months. The starting dose was the dose causing the inhibition of 50% of cancer cell growth (IC_50_). The drug dose was progressively increased to 1 μg/ml in approximately 2 months, to 5 μg/ml after other 2 months and, finally, to 10 μg/ml after additional 2 months. The established MEKi resistant HCT116 and LIM1215 cancer cell lines (HCT116-MR and LIM1215-MR) were then maintained in continuous culture with this maximally achieved dose of MEKi that allowed cellular proliferation.

SW48-MR and LIM1215-MR cells were grown in RPMI-1640 (Lonza) supplemented with 10% FBS, 1% penicillin/streptomycin. HCT116-MR cell line was grown in McCoy culture medium (Lonza, Cologne, Germany), supplemented with 10% FBS (Lonza), 1% penicillin/streptomycin (Lonza).

C57BL/6 and BALB/c immune-competent mice were respectively injected with MC38 and CT26 colon cancer syngeneic cell lines. When established tumors of approximately 200–300 mm^3^ in diameter were detected, mice were treated continuously by oral gavage injection of MEKi daily (BAY86–9766, 5 mg/kg). When tumors were resuming growth despite MEKi treatment, MEKi resistant tumors were surgically removed and homogenized into single-cell suspensions used to generate in vitro MC38 and CT26 MEKi resistant cell lines as described above. MC38-MR cell line was grown in DMEM (Lonza) supplemented with 10% FBS, 1% penicillin/streptomycin (Lonza). CT26-MR cell line was grown in RPMI-1640 (Lonza) supplemented with 10% FBS, 1% penicillin/streptomycin (Lonza).

### Microarray gene expression analysis

Agilent (Agilent Technologies, Waldbronn, Germany) microarray analyses were performed to assess baseline gene expression profile for SW48 and SW48-MR colon cancer cells using a one color labeling microarray system. The absolute amount and purity (A260/280 nm ratio) of total RNA samples were determined by spectrophotometry (Nanodrop, Thermofisher) and the size distribution was assessed by Agilent Bioanalyzer. Eight hundred nanograms of total RNA were converted into labelled cRNA with nucleotides coupled to a fluorescent dye (either Cy3 or Cy5) following the manufacturer’s protocol (Quick Amp Kit, Agilent). Yield and purity (A260/280 nm ratio) of cRNAs were determined by spectrophotometry (Nanodrop, Thermofisher). 825 ng of cRNA-labeled from SW48 and SW48-MR colon cancer cell lines were hybridized to Agilent Human Whole Genome 4 × 44 k Microarrays. Data were extracted from slide image using Agilent Feature Extraction software (v.10.5). The raw data and associated sample information were loaded and processed by Gene Spring® 11.5X (Agilent Technologies). For identification of genes significantly altered in resistant cells, total detected entities were filtered by signal intensity value (upper cut-off 100th and lower cut-off 20th percentile) and flag to remove very low signal entities. Experiments were performed in triplicate and data were analyzed using Student’s t test (*p* < 0.05) with a Benjamani-Hochberg multiple test correction to minimize selection of false positives. Of the significantly differentially expressed RNA, only those with greater than 2-fold increase or 2-fold decrease as compared to the controls were used for further analysis. Functional and network analyses of statistically significant gene expression changes were performed using Ingenuity Pathways Analysis (IPA) 8.0 (Ingenuity® Systems, http://www.ingenuity.com). Analysis considered all genes from the data set that met the 2-fold (*p*-value < 0.05) change cut-off and that were associated with biological functions in the Ingenuity Pathways Knowledge Base. The significance of the association between the data set and the canonical pathway was measured in 2 ways: 1- Ratio of the number of genes from the dataset that map to the pathway divided by the total number of genes that map to the canonical pathway is displayed; 2- Fisher’s exact test was used to calculate a *p* value determining the probability that the association between the genes in the dataset and the canonical pathway is explained by chance alone.

### MTT assay

HCT116, HCT116-MR, LIM1215 and LIM1215-MR cells were seeded into 24-well plates (1 × 10^4^ cells per well) and were treated with different doses of drugs for 96 h. Cell proliferation was measured with the 3-(4, 5-dimethylthiazol-2-yl)-2, 5- diphenyltetrazolium bromide (MTT) (Sigma) assay (final concentration, 5 mg/mL-Sigma-Aldrich). The MTT solution was removed and remained formazan crystals were extracted with Isopropanol supplemented 1% HCl (200 μl/well). The 24-well were shaker for 10 min then 100 μl was subsequently transferred to 96-well. Absorbance of the formazan’s solution in Isopropanol-HCl was measured spectrophotometrically at a wavelength of 550 nm. The IC50 value was determined by interpolation from the dose-response curves. Results represent the median of three separate experiments, each performed in triplicate.

### RNA extraction and qRT-PCR

Total RNA was prepared using TRIzol reagent (Life Technologies) and reverse-transcribed into cDNA by SensiFast reverse transcriptase (Bioline) according to the manufacturer instruction. Expression levels of genes encoding for STAT3, PD-L1 and EGFR were analyzed using Real Time quantitative PCR. Amplification was conducted using the SYBER Green PCR Master Mix (Applied Biosystems). All samples were run in duplicate using a Quant studio 7 Flex (Applied Biosystem) and the expression levels of target genes were standardized by housekeeping gene 18S using the 2^-ΔΔCt^ method.

### RNA interference

The small inhibitor duplex RNAs (siRNA) (ON-target plus SMARTpool) siSTAT3 (human: # L-003544-00-000) and siCD274 (human: #L-015836-01-000) were from Dharmacon (Lafayette, CO). The siCONTROL Non-Targeting Pool (#D-001206-13-05) was used as a negative (scrambled) control. Cells were transfected with 100 nM siRNAs using Dharmafect reagent following manufacturer’s instructions. The day before transfection, the cells were plated in 35 mm dishes at 40% of confluence in medium supplemented with 5% FBS without antibiotics. Cells were harvested 48 h after transfection. PCR for STAT3 and PD-L1 expression was done. RNA extraction was performed by the RNeasy Kit (Qiagen, Crawley, West Sussex, UK) following manufacturer’s instructions. The RNA was quantified by Nanodrop (Thermo Scientific, Wilmington, DE) and RNA integrity was analyzed by the 2100 Bioanalyzer (Agilent Technologies).

### Western blot analysis

Western blot analysis was performed as previously described [10, 11]. The protein concentration was determined using a Bradford assay (Bio-Rad) and equal amounts of proteins were separated by SDS-PAGE gel and transferred to nitrocellulose membrane (Bio-Rad). The membranes were probed with primary antibodies followed by incubation with HRP-conjugated secondary antibodies. The following antibodies: EGFR monoclonal antibody (#4267), pEGFR monoclonal antibody (#3777), E-cadherin, monoclonal antibody (#3195), STAT3 monoclonal antibody (#4904), pSTAT3 monoclonal antibody(#9145), AKT policlonal antibody (#9272), pAKT monoclonal antibody (#4060), Vimentin monoclonal antibody (#5741), PD-L1 monoclonal antibody (#13684), p44/42 MAPK polyclonal antibody (#9102), phospho-p44/42MAPK monoclonal antibody (#9106), MEK 1/2 monoclonal antibody(#4694), pMEK1/2 monoclonal antibody (#9154), Cleaved Caspase 9 antibody (#9505), Caspase 3 antibody (#9662), SNAIL monoclonal antibody (#3879), SLUG monoclonal antibody (#9585) and Bcl-2 monoclonal antibody (#4223) were from Cell Signaling (Beverly, MA, USA). Mouse PD-L1 antibody (#ABM4E54) was for Abcam (Cambridge, UK). Monoclonal anti-α-tubulin antibody (T8203) was from Sigma Chemical Co. (St. Louis, MO, USA). Secondary antibodies goat anti-rabbit IgG and rabbit anti-mouse IgG were from Bio-rad (Hercules, CA, USA). Immunoreactive proteins were visualized by enhanced chemiluminescence (ECL plus, Thermo Fisher Scientific, Rockford, IL, USA). Each experiment was done in triplicate.

### Immunofluorescence analysis

The cells were fixed with 4% paraformaldehyde for 10 min, permeabilized in 0.5% Triton X-100 for 10 min and blocked in PBS supplemented with 3% BSA for 30 min. After each step, the cells were rinsed in PBS, incubated for 1 h at room temperature with primary antibodies anti-EGF Receptor (Abcam), anti-PD-L1(Cell signaling) followed by incubation with secondary antibodies Alexa Fluor 488 goat anti-mouse IgG (ThermoFisher) or Alexa Fluor 532 goat anti-rabbit IgG (ThermoFisher) for 30 min at RT. Finally, the coverslips were incubated for 15 min at 37 °C with 4′,6-Diamidino-2-phenylindole dihydrochloride (DAPI) and then examined under the fluorescence confocal microscope Zeiss LSM 700 (Zeiss, Oberkochen, Germany).

### Nuclei isolation

Cells were lysed in Buffer B2 (Hepes pH 7.9 10 mM, EDTA 1 mM, KCl 60 mM, DTT 1 mM) supplemented with protease inhibitor, phosphatase inhibitor and 0.2% NP-40. Centrifugation at 3000×g for 15 min and the supernatant was centrifuged further at 13.000×g for 15 min to obtain the cytosolic fraction. The nuclear pellet was resuspended in buffer containing 60% sucrose and separated by centrifugation at 6000×g for 10 min. Nuclei were dissolved in Buffer B4 (Tris-HCl pH 7.8250 mM, KCl 60 mM, DTT 1 mM) supplemented with protease inhibitor and phosphatase inhibitor. Nuclei were dissolved using alternated temperatures (− 80 °C and 37 °C) and clarified by centrifugation at 9500×g for 10 min.

### Mouse experiments

C57BL/6 N mice and BALB/c mice from Charles River were maintained in the animal facilities of MDACC following standard animal regulation and strict health controls. 1 × 10^5^ MC38-MR and 3 × 10^5^ CT26-MR cells were suspended in 200 μl of Matrigel (BD Biosciences, Milan, IT): PBS (1:1) and were subcutaneously injected to the right flank of C57BL/6 and BALB/c mice respectively. Two weeks after injections when tumors were between 200 and 300 mm^3^, mice were randomly assigned to one of the following groups (ten mice per group): group 1: vehicle, administrated intraperitoneally (i.p.); group 2: MEKi (BAY86–9766 25 mg/kg every day for 5 days a week, by oral gavage); Group 3: EGFRi (Erltonib 10 mg/Kg very day for 5 days a week, by oral gavage); Group 4: PD-L1i (clone 10F.9G2 injected twice a week i.p. at total dose of 200 μg/mouse); Group 5: MEKi plus PD-L1i at same doses mentioned above; Group 6: MEKi plus PD-L1i plus EGFRi at same doses mentioned above. The treatment was continued for 3 weeks and mice were followed for additional 5 weeks after end of treatment. When tumors reached approximately 2.000 mm^3^ mice were euthanized. In all experiments, mice body weights were monitored daily. Tumor size was evaluated twice a week by calliper measurements using the following formula: π/6 × larger diameter × (smaller diameter)^2^. For assessment of tumor response to treatment, we used volume measurements and adopted a classification methodology loosely inspired by clinical criteria: (i) tumor regression (or shrinkage) was defined as a decrease of at least 50% in the volume of target lesions, taking as reference the baseline tumor volume; (ii) at least a 35% increase in tumor volume identified disease progression; and (iii) responses that were neither sufficient reduction to qualify for shrinkage or sufficient increase to qualify for progression were considered as disease stabilization.

### Statistical analysis

Results were expressed as mean ± SD, and n refers to the number of sample replicates. The statistical differences between the means were determined by one-way ANOVA followed by Tukey’s multiple comparison tests with Prism software (version 6.01; GraphPad, USA). *p* < 0.05 was considered to be statistically significant. The false discovery rate (FDR) was applied as a multiple test correction method. Average gene expression values between experimental groups were compared (on log scale) by means of a modified ANOVA (*p* < 0.05). Genes in the resistant group were identified as being differentially expressed compared to parental cells if they had a fold-change (FC) in expression of at least 3 and a *p*-value < 0.05.

## Results

### Human colon cancer cells with acquired resistance to MEKi display a CMS4 gene expression profile

**Fig. 1:**
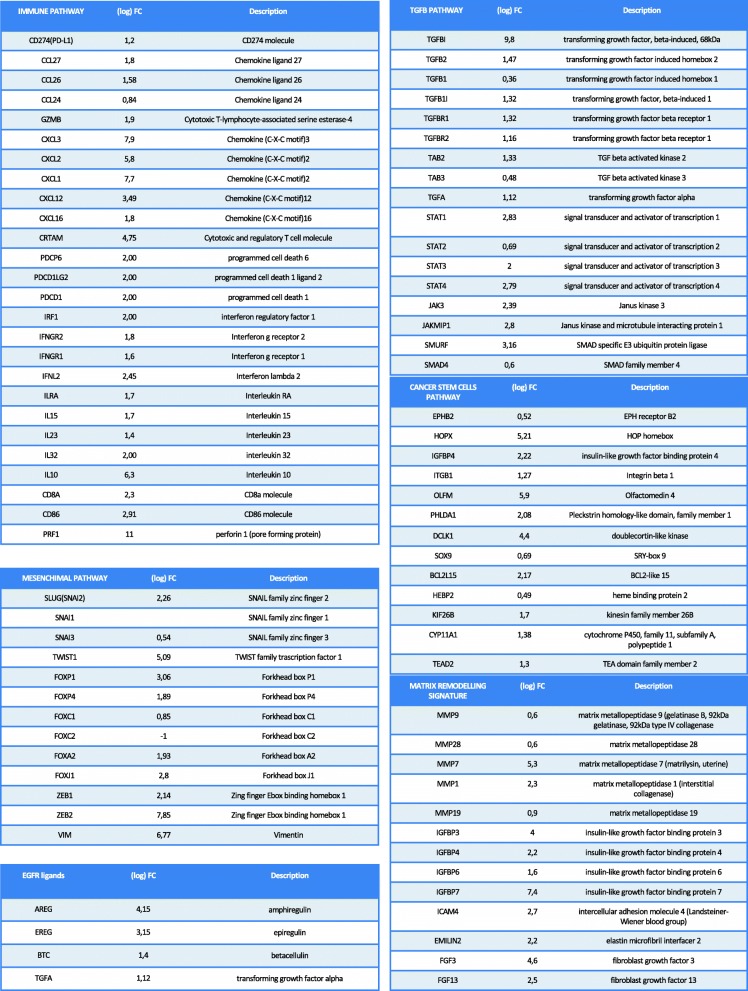
CMS4 gene expression signature in a preclinical model of colon cancer cells with acquired resistance to MEKi. Agilent microarray analyses were performed to assess baseline gene expression profile for SW48 and SW48-MR Colon cancer cell lines, as described in Materials and Methods. (Log) fold change indicates relative microRNA expression levels in SW48-MR respect to SW48 cells. The genes whose expression is up regulated in SW48-MR compared to SW48 cells are represented in schematic tables. These genes are involved in CMS4 gene signature

In previous works, we have demonstrated that MEK is a key downstream effector of EGFR pathway that must be inhibit to prevent and/or delay the onset of acquired resistance to anti-EGFR treatment [3, 10, 11, 18–21]. Nevertheless, we have found that some tumors after an initial benefit to MEKi treatment, started to regrowth limiting its use [11]. In order to understand the mechanism underlying MEKi resistance, we have generated MEKi resistant cell line, SW48-MR as described in Material and Methods. To investigate the potential molecular pathways involved in MEKi-resistance mRNAs from SW48 and SW48-MR cells were extracted and assessed for global gene expression changes by microarray analysis (Fig. 1). We conducted gene set enrichment analyses (GSEA) using previously described signatures of pathway activity and well-characterized cellular processes to characterize the molecular pathways specific for this MEKi resistant model. Among the genes that were up-regulated in SW48-MR versus SW48 cells we have identified several genes involved in the PD-L1 pathway. In particular *PD-L1* gene was up-regulated in the resistant cells compare to parental cells (Fig. 1). Moreover, genes overexpressed in MEKi resistant cells were functionally related in pathways involving immune cell activation, inflammation, and antigen processing and presentation such as Programmed Cell Death 1 *(PDCD1),* CD86 molecule *(CD86),* CD8 molecule *(CD8),* Pore Forming protein 1 *(Perforin 1),* Interferon Regulatory Factor 1 *(IRF1)*, Cytotoxic and regulatory T cell molecule *(CRTAM)* (Fig. 1). Moreover our analysis revealed an increase levels of genes known to be involved in mesenchymal progression, EMT transition and in matrix modeling such as Transforming Growth Factor beta 1 (*TGFB1*), Transforming Growth Factor beta activated kinase 2 *(TAB2),* Transforming Growth Factor beta kinase 3 *(TAB3),* Transforming Growth Factor alpha *(TGFA),* Signal Transducer and Activator of Trascription 1 *(STAT1),* Signal Transducer and Activator of Trascription 2 *(STAT2),* Signal Transducer and Activator of Trascription 3 *(STAT3),* Signal Transducer and Activator of Trascription 4 *(STAT4),* Janus kinase 3 *(JAK3),* SMAD specific E3 ubiquitin protein ligase *(SMURF),* SMAD family member *(SMAD4),* Twist homolog 1 (Drosophila) (*TWIST1*), Vimentin (*VIM*), SNAIL family zinc Finger 1 *(SNAI1),* SNAIL family zinc Finger 2 *(SLUG),* SNAIL family zinc Finger 3 *(SNAI3),* Matrix Metallo Peptidase 9, 28, 7, 1, 19 *(MMP9, MMP28, MMP7, MMP1, MMP19),* intercellular adhesion molecule 4 *(ICAM4),* elastin microbril interfacer 2 *(EMILIN2).* These results demonstrate an enhanced immune-reactive microenvironment in MEKi resistant cells compared with parental ones. In fact, high expression of genes specific to Treg cells, myeloid-derived suppressor cells (MDSCs), monocyte-derived cells and TH17 cells, present in our MEKi resistant tumor are typically seen in the microenvironment of immune-tolerant malignancies (Fig. 1). This ‘inflamed’ immune profile was similar to CMS4 subtype of CRC that is characterized by marked up-regulation of immune suppressive factors, such as Transforming Growth Factor Receptor beta (*TGFβ)* and Chemokine (C-X-C motif)12 (*CXCL12)*, and high expression of genes encoding chemokines that attract myeloid cells, including C-C motif chemokine ligand 2 *(CCL2)* and the related cytokines *IL-23* and IL-17 [14–17]. Moreover, CMS4 tumors were characterized by activation of pathways related to EMT and steaminess, such as TGFβ pathway members and integrin, and showed marked overexpression of proteins implicated in extracellular matrix remodeling and complement signaling [14–17]. All the genes correlated to these signatures were overexpressed in our MEKi resistant cells, confirming the correlation between our MEKi resistant cell lines and CMS4 tumors (Fig. 1).

Other less well-known genes involved in these processes were also up-regulated: Platelet-derived Growth Factor Receptor (*PDGFRB*), Fibroblast Growth Factor 3 (*FGF3*), Fibroblast Growth Factor 13 (*FGF13*), Insulin-like growth factor binding protein 3 *(IGFBP3*), Insulin-like growth factor binding protein 7 *(IGFBP7).* In addition, all EGFR ligands are up-regulated in the resistant cells compared to parental ones, underlying a role of EGFR activation in MEKi resistance landscape (Fig. 1).

**Fig. 2:**
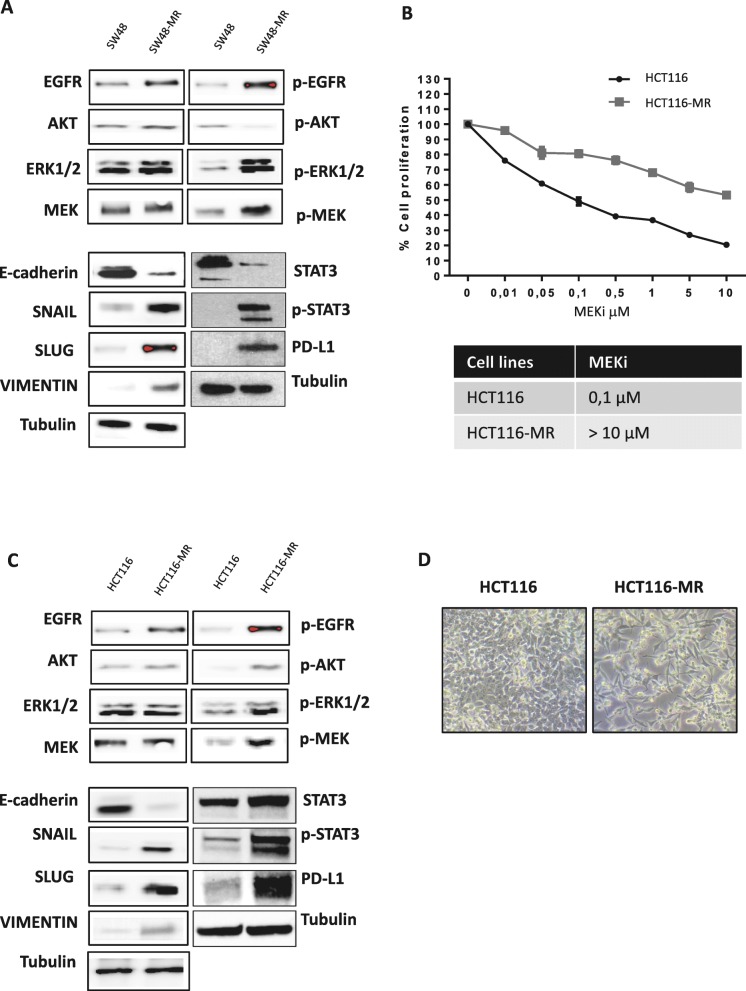
Establishment and characterization of MEKi-resistant human colorectal cancer cell lines. **a-c.** Total cell protein extracts (50 μg) were subjected to immunoblotting with the indicated antibodies, as described in Materials and Methods. Anti-tubulin antibody was used for normalization of protein extract content. Experiments were repeated three times. **b.** HCT116 and HCT116-MR Cells were treated with different concentrations of BAY86–9766 (0.01–10 μM) for 96 h and evaluated for proliferation by MTT staining, as described in Materials and Methods. The results are the average ± SD of three independent experiments each done in triplicate. **d.** Morphologic change in resistant cell lines compared to parental cell lines

To further expand these observations, we have selected other two CRC cell lines sensitive to MEK blockade, such as HCT116 (*KRAS* G13D) and LIM1215 (all *RAS* WT), to generate by an in vitro selection new models of acquired resistance to MEKi (Fig. 2, Additional file 1: Figure S1). After the establishment of MEKi resistant cell lines we have characterized the resistant phenotype by cell proliferation analysis using a 3-(4,5-dimethylthiazol-2-yl)-2,5- diphenyltetrazolium bromide (MTT) and by 5-bromo-2-deoxyuridine (BrdU) incorporation assay, in the presence of MEKi. To assess whether gene expression–based subtypes are recapitulated at the protein level, we performed a western blot analysis and we found that all the pathways that are up regulated in CMS4 subtype were up-regulated in these resistant cell lines (Fig. 2, Additional file 1: Figure S1). As depicted in Fig. 2, moving forward the MEKi phenotype we assisted to the loss of epithelial features and acquisition of mesenchymal markers and morphology. In particular, we showed an up-regulation of SLUG, SNAIL and Vimentin and down regulation of E-cadherin in the resistant cells compared to parental ones. Moreover, this change in the morphology is accompanied by up-regulation of PD-L1 expression and activation of EGFR and its downstream pathway in the resistant models (Fig. 2, Additional file 1: Figure S1).

### STAT3 promotes PD-L1 expression in colon cancer models with acquired resistance to MEKi which can be enhanced via cooperation of EGFR

**Fig. 3:**
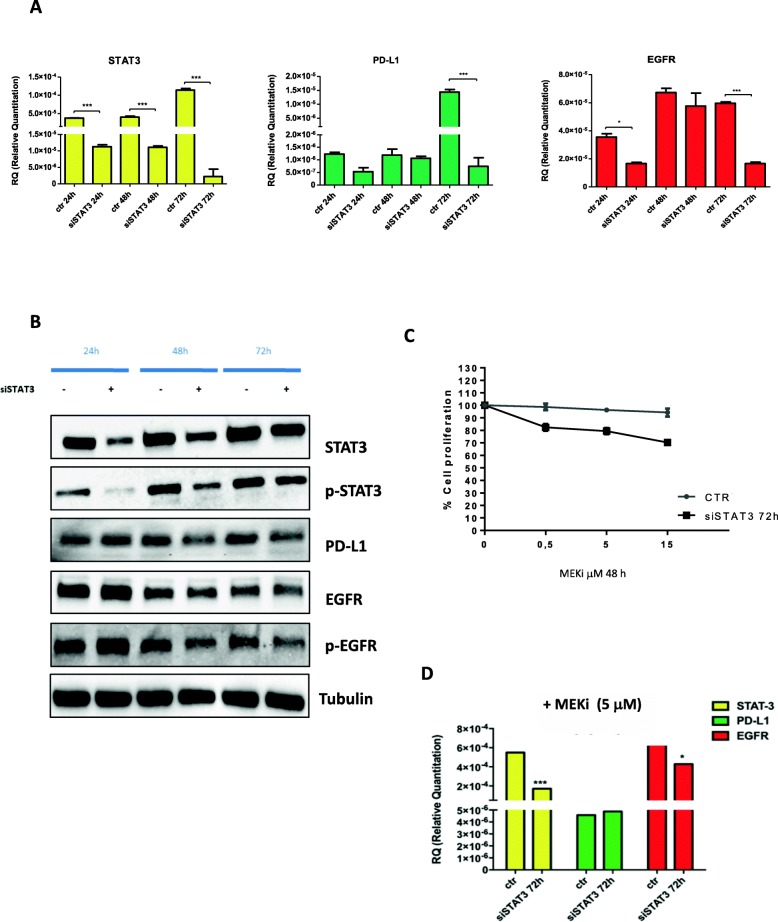
Effect of STAT3 inhibition on PD-L1 and EGFR expression in HCT116-MR colon cancer cells. **a.** The mRNA expression levels of STAT3, PD-L1 and EGFR 24 h, 48 h, and 72 h after transfection with a specific siRNA targeting STAT3 in HCT116-MR cells. One way anova test: *p < 0.05, ***p < 0.001 compared with the control. **b.** Western blot analysis of cell signalling proteins in HCT116-MR cells transfected with a specific siRNA targeting STAT3 or with a scrambled, control siRNA for 24, 48 and 72 h. Total cell protein extracts were subjected to immune-blotting with the indicated antibodies, as described in Materials and Methods. **c.** HCT116-MR cells were transfected with siRNA targeting STAT3. 72 h after transfection, cells were treated with increasing concentrations of MEKi **(**BAY86–9766, range 0–15 μM). Cells were evaluated for proliferation after 48 h by MTT staining. The results are average ± SD of three independent experiments each done in duplicate. **d.** The mRNA expression levels of STAT3, PD-L1 and EGFR with a specific siRNA targeting STAT3 or with a scrambled, control siRNA for 72 h and subsequently treated with the indicated dose of MEKi **(**BAY86–9766) treatment for 24 h. One way anova test: *p < 0.05, ***p < 0.001 compared with the control

**Fig. 4:**
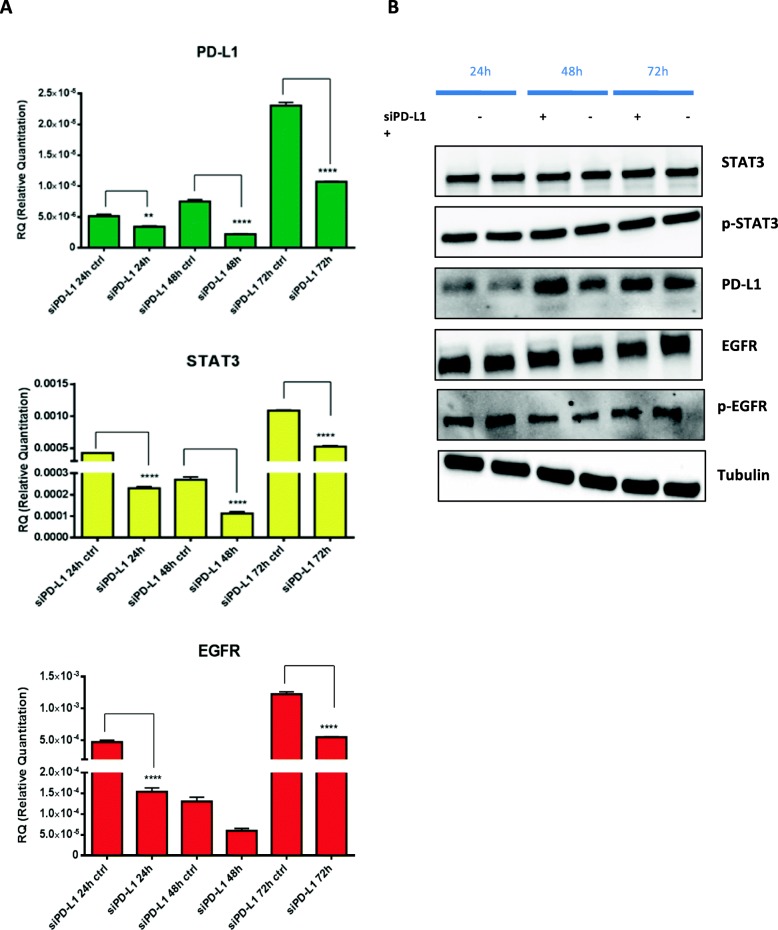
Effect of PD-L1 inhibition on STAT3 and EGFR expression in HCT116-MR colon cancer cells. **a**. The mRNA expression levels of PD-L1, STAT3 and EGFR at 24 h, 48 h, and 72 h after transfection with a specific siRNA targeting PD-L1. One way anova test: **p < 0.002; ****p < 0.0001 compared with the control. **b.** Western blot analysis of cell signalling proteins in HCT116-MR cells transfected with a specific siRNA targeting PD-L1 or with a scrambled, control siRNA for 24, 48 and 72 h. Total cell protein extracts were subjected to immune-blotting with the indicated antibodies, as described in Materials and Methods

Understanding molecular mechanisms that modulate PD-L1 expression would likely lead to improve treatments for patients. The regulation of PD-L1 expression is likely complex and is regulated by multiple signaling pathways, some that have been investigated including the JAK/STAT (Janus kinase signal transducer and activation of transcription) [21, 22]. Since in our MEKi resistant model we have found an up-regulation of *STAT3* gene, we investigated the mechanism of its activation (Fig. 1). *STAT3* can be activated by several secreted factors, cytokines and different tyrosine kinases receptors (TKRs) including EGFR activation [23]. To evaluate the role of STAT3 in the PD-L1 modulation and subsequently in mediating MEKi resistance and to understand its correlation with EGFR we have first modulated the expression of STAT3 by using a siRNA technology in the MEKi resistant cell line (HCT116-MR), and then we have assessed the PD-L1 and EGFR expression by performing western blot analysis (Fig. 3 a-b). Transfection with a specific STAT3 siRNA significantly reduced PDL1 and EGFR mRNA and protein expression in HCT116-MR cells at 72 and 48 h respectively, as shown in Fig. 3 a-b. These data confirmed a direct correlation between STAT3, EGFR and PD-L1 expression. Moreover, STAT3 silencing also restored the sensitivity of HCT116-MR to the inhibitor effect of MEKi, as shown in Fig. 3 c-d. To further determine if PD-L1 activation could be involved in the acquisition of MEKi resistance in HCT116-MR cells, we investigated whether reduction of PD-L1 expression could modulate STAT3 and EGFR expression (Fig. 4). The inhibition of PD-L1 expression by siRNA determined a significant reduction of STAT3 and EGFR expression, underlying the strong correlations among these three factors and their potential role in the development of resistance to MEKi (Fig. 4).

### Nuclear expression of PD-L1 in MEKi resistant colon cancer cells

PD-L1 is generally a membrane protein and its localization could change in different caner type [24]. Several groups have already demonstrated that mislocalization of it in the nucleus could promote drug resistance and is associated with short survival durations [24, 25].

**Fig. 5:**
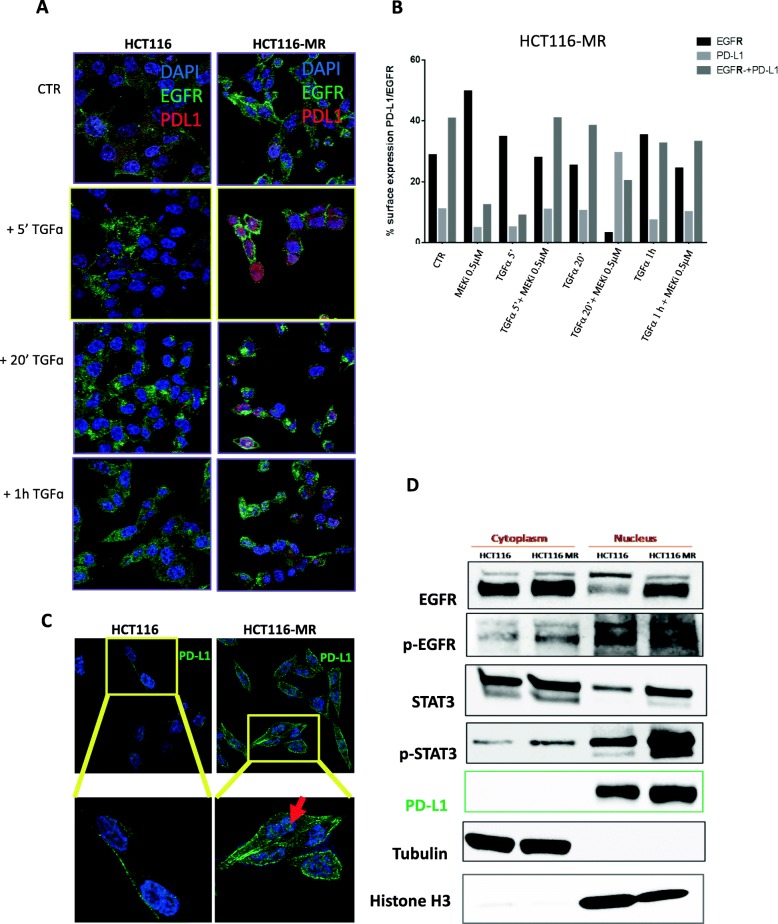
Expression of PD-L1 and EGFR by immunofluorescence analysis. **a.** Immunofluorescence analysis of the localization of EGFR and PD-L1 in HCT116 and HCT116-MR cells after exposure to different time of TGFα (5 min, 20 min and 1 h). Cells were stained with anti-EGFR antibody followed by secondary antibody labeled with Alexa Fluor 488 and anti-PD-L1 antibody followed by secondary antibody Alexa Fluor 532. DAPI was included to stain the nucleus. **b.** Cytofluorimetric analysis of HCT116 MR cells. Cells are stimulated with TGFα for 5 min, 20 min and 1 h alone or in combination with MEKi (BAY86–9766 0,5 μM) and then we investigated the presence of EGFR and PD-L1 on the surface of cells. **c.** An upregulation of PD-L1 is observed in the nucleus of HCT116 MR cells. Green color from Alexa Fluor 488 for PD-L1 and blue color from DAPI for visualization of nuclei. **d.** HCT116 and HCT116-MR were lysed to obtain cytosolic and nuclear fragments that were analyzed by Western blot analysis. Tubulin and Histone H3 antibodies were used as loading and purity control of the cytosolic and nuclear fractions, respectively

With this aim, to determine the location of the PD-L1 and EGFR protein in our resistant models, we observed the staining pattern of anti-PD-L1 and anti-EGFR affinity-purified antibodies by indirect immunofluorescence. HCT116 and HCT116-MR cells were plated on slides, serum starved overnight and then treated with TGFα at different time point (5 min, 20 min and 1 h). As depicted in Fig. 5 a, in HCT116-MR cells treatment with TGFα increased the expression of PD-L1 in the nucleus, this effect is more evident at 5 min time point (Fig. 5a). To better characterize this result, we studied the distribution of PD-L1 and EGFR on the surface cells by flow cytometry in HCT116-MR cells. According to immunofluorescence staining after TGFα treatment and also after treatment with MEKi at dose of 0,5 μM the expression of PD-L1 on cell-surface decreased as well as expression of both PD-L1 and EGFR on cell-surface compared to control cells (Fig. 5b). The same observations were made using confocal microscopy after Z-stacks images; PD-L1 is more expressed in the HCT116-MR cells on the cell surface but above all the presence of the protein is evident in the nucleus (Fig. 5c).

To confirm the presence of PD-L1 in nuclear compartment, nuclear and cytoplasmic extracts were performed in HCT116 and HCT116-MR cell line. Western blot analysis revealed that PD-L1 was predominantly in the nuclear fraction, as also p-EGFR and p-STAT3. Histone H3 and tubulin were used as nuclear and cytoplasmic controls, respectively (Fig. 5d).

Based on these findings, PD-L1 expression in MEKi resistant cells is more evident in the cell nuclear compartment. This effect could explain also anti-apoptotic activity present in the MEKi resistant cell line compared to parental one. We tested expression of the anti-apoptotic components by Western blot analysis. As shown in Additional file 1: Figure S1B, we observed a strong increasing in expression levels of BcL2 in HCT116-MR cells, suggesting a probable activation of anti apoptotic mechanisms in this resistant cell line.

### Effects of combined treatment of MEK inhibitor, EGFR inhibitor and PD-L1 inhibitor on syngeneic colorectal cancer tumor xenograft models with acquired resistance to MEKi

**Fig. 6:**
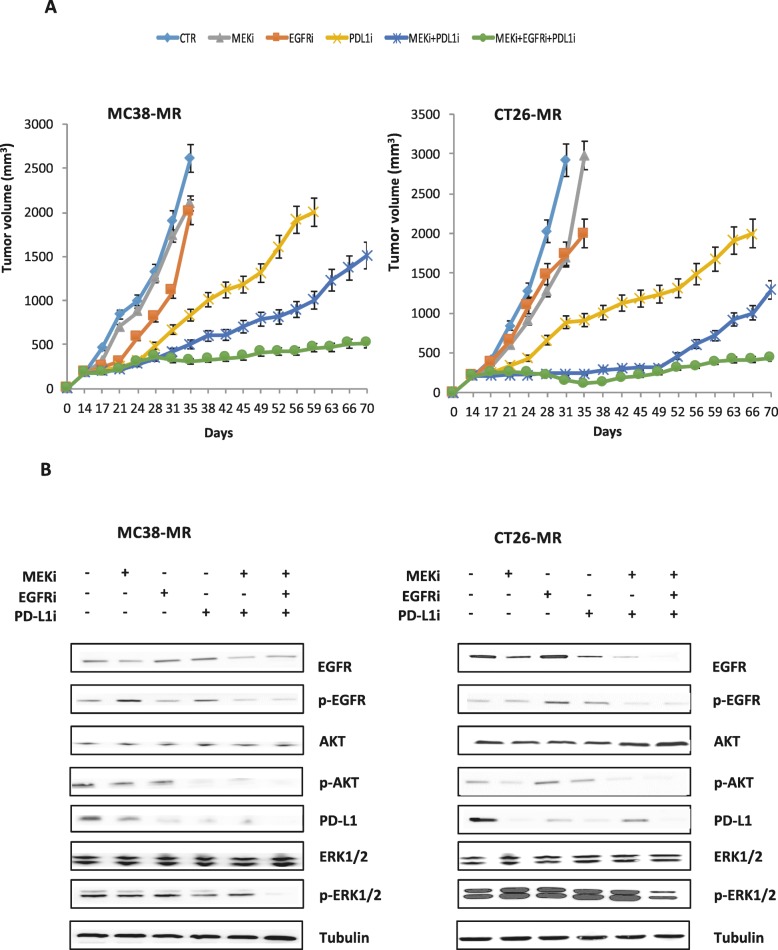
Effects of combined treatment of MEK inhibitor, EGFR inhibitor and PD-L1 inhibitor on syngeneic colorectal cancer tumor xenograft models with acquired resistance to MEKi. **a.** C57BL/6 and BALB/c immune-competent mice were injected subcutaneously in the right flank with MC38-MR and CT26-MR cells as described in the Materials and Methods. After two weeks (average tumor size 200–300 mm^3^) mice were treated with: PBS (phosphate-buffered saline) as control, MEKi (BAY86–9766 25 mg/kg every day for 5 days a week, by oral gavage), EGFRi (erlotinib 10 mg/Kg every day for 5 days a week by oral gavage), mouse PD-L1i (clone 10F.9G2) injected twice a week i.p. at total dose of 200 μg/mouse, MEKi+PD-L1i at doses described above and MEKi+EGFRi+PD-L1i at doses described above. The treatment was continued for 3 weeks. Mice were followed for additional 5 weeks. Each group consisted of 10 mice. The mean data are present. Tumor growth curves were calculated based on twice weekly times tumor measurements during the treatment period and after 5 weeks of observation after termination of therapy. Animals were sacrificed when tumors achieved 2.000 mm^3^ in size. **b.** At the end of treatment one mouse per group treated with MEKi, EGFRi, PD-L1i or with their combination was sacrificed. As control, we used one mouse engraft with MC38-MR and CT26-MR respectively that has not undergone to any type of treatment. Tumor samples were collected and total cell protein extracts were subjected to immunoblotting with the indicated antibodies, as described in Materials and Methods. Anti-tubulin antibody was used for normalization of protein extract content

To extend these in vitro findings, we performed an in vivo study using MC38 and CT26 MEKi resistant syngeneic models. MC38 (all *RAS* WT) and CT26 (*KRA*S G12D) are the only two syngeneic colon cancer cells available. These two cell lines should recapitulate the whole scenario of acquired resistance mechanisms to MEKi in both WT and *RAS* mutated contest. We generated MEKi resistant cells starting from MEKi sensitive MC38 and CT26 syngeneic colon cancer cells, which were grown as tumor xenografts in in vivo C57BL/6 and BALB/c immune-competent mice that were exposed to continuous treatment with an optimal therapeutic dose of the drug. Continuous treatment caused tumor growth suppression within the first weeks of treatment (Additional file 2: Figure S2A). However, after approximately 12 weeks, in most mice tumors resumed growth despite MEKi treatment, reaching a growth rate comparable to untreated control MC38 and CT26 tumors within 18–20 weeks (Additional file 2: Figure S2A). MEKi resistant tumors were surgically removed and homogenized into single-cell suspensions used to generate in vitro MC38 and CT26 MEKi resistant cell lines, which displayed resistance to the growth inhibitory effects of MEKi treatment (Additional file 2: Figure S2B). These models are a valuable tool to provide additional information on the potential for clinical activity of immune therapies, and could also improve the understanding of immune response in mCRC. MC38 and CT26 MEKi resistant colon cancer cells have been injected subcutaneously in C57BL/6 and BALB/c mice respectively. Mice have been treated with MEKi, EGFR inhibitor (EGFRi), murine PD-L1 inhibitor (PD-L1i) and their combination (Fig. 6a). We used erlotinib a tyrosine kinase inhibitor as EGFRi that is able to recognize and inhibit the mouse EGFR. Treatments were started 2 weeks after the injection of tumor cells and lasted for 3 weeks. After the end of treatment mice were followed for additional 5 weeks. At the end of 3 weeks treatment, all mice treated with vehicle, MEKi or EGFRi alone reached the maximum allowed tumor size of 2000 mm^3^. Among the single agent treatments, the group treated with PD-L1i showed the greatest tumor growth inhibition in both xenografts. As shown in Figs. 6a, both combined treatment regimens markedly suppressed tumor growth compared with vehicle, MEKi alone, and EGFRi alone. Combined treatment of MEKi, EGFRi and PD-L1i resulted in a marked inhibition of tumor growth in both MC38–MR and CT26-MR xenograft models. Indeed, at the end of treatment period, the triplet combination treatment caused a more evident synergistic effect, with no palpable tumor present in 4 out of 10 mice engrafted with MC38-MR cells and 3 out of 10 mice engrafted with CT26-MR cells (Fig. 6a). The synergistic effect of triplet combination treatment was also more marked at the end of observation period elucidating the long-term effect of this combination. This project reveals a strategy to potentially improve the efficacy of MEK inhibition by co-treatment with EGFR and PD-L1 inhibitors via modulation of host immune responses.

### Effects of MEKi plus EGFRi plus PD-L1i treatment on EGFR-dependent intracellular signaling pathways in the mouse MC38-MR and CT26-MR syngeneic xenograft models

To understand whether the synergistic anti-tumor activity obtained by the combined treatment with MEKi, EGFRi and PD-L1i was due to a more effective inhibition of key intracellular signals for cell survival and proliferation, tumors were collected at the end of the treatment from mice engrafted with both MC38-MR and CT26-MR cell lines. One mouse per arm was sacrificed from MEKi, EGFRi, PD-L1i, MEKi plus PD-L1i and MEKi plus PD-L1 plus EGFRi treatment groups. As control, we used one mouse engrafted with both resistant cell lines that has not undergone any type of treatment. First, we assessed the phosphorylation status of EGFR and its downstream effectors AKT and MAPK by western blot analysis. As shown in Fig. 6b, in tumor specimens treated with MEKi EGFRi and PD-L1i alone no change or partially inhibition in expression of pEGFR, pAKT and pMAPK was observed. The combined treatment with MEKi and PD-L1i substantially inhibited phosphorylation of EGFR, MAPK and AKT compared to single agent treatments (Fig. 6b). In particular the expression of pMAPK was almost completely ablated only in the triplet treatment arm in both MC38-MR and CT26-MR models underling the synergistic effect of this combination. Moreover, also PD-L1 expression was totally abolished only in the triplet treatment group while no change or partial inhibition of its expression was observed in the other treatment group.

## Discussion

Despite major advances in technologies to monitor complex molecular profiles of cancer and the rapidly expanding repertoire of treatment options, including immunotherapy, translating molecular observations into treatment decisions remains challenging in routine patient care due to cancer heterogeneity [3, 9, 17, 26]. CRC in particular represents a heterogeneous group of dynamic diseases with differing sets of genetic events, accompanying immune response, and influences of exogenous factors, providing a challenge for personalized therapeutic approaches [5, 17]. Several attempts have been made to identify molecular gene signatures that can help to classify CRC in different subtypes with specific clinical and therapeutic relevance [27–34]. Recently, efforts have been made by the Colorectal Cancer Subtyping Consortium (CRCSC) in order to capture the main overall gene expression variability for this tumor type in a unique classification system [15]. This has led to distinguish four Consensus Molecular Subtypes (CMS): CMS1 [characterized by microsatellite instability (MSI) and immune patterns], CMS2 (chromosomal instability and WNT activation), CMS3 (metabolic pattern), and CMS4 [epithelial–mesenchymal transition (EMT)] [15].

Moreover, in the past few years, the rapidly advancing field of cancer immunology has produced several new methods of treating cancer, that increase the strength of immune responses against tumors [35]. Immune classification of cancers has shed new light in patients’ care providing prognostic and predictive factors for chemotherapies and immunotherapies, such as immune checkpoint inhibitors (14, 16, 17). Unfortunately, none of the several treatments tested proved a real beneficial effect in CRC. Strikingly positive effects have been seen only in patients with MSI tumors [26, 36–38]. However, not all CRC patients with high mutational load respond to immune checkpoint inhibitor therapy, suggesting that the cancer cells may have evolved alternate mechanisms to evade immune recognition prior to treatment. In addition, many patients who initially respond develop then resistance mechanisms [26].

In this scenario, new biomarkers are needed and a better selection of CRC patients that could respond to immunotherapies are of substantial interest, particularly considering recent checkpoint blockade failures in CRC. Interestingly, molecular characterization of CRC has shown that CMS are associated with specific immune infiltration profiles corresponding with characteristic mechanisms of immune escape [14, 39]. In particular, the CMS4 subtype presents high immune infiltrate but with an unfavorable, inflamed molecular orientation characterized by intratumoral MDSC, M2-macrophages and B-cells associated with pro-inflammatory gene expression, including myeloid chemokines, immune suppressive molecules and complement factors [14, 16, 17]. Theoretically, immunotherapy could be useful for all CRC if it is possible to convert the tumor towards a “CMS1 like” immune phenotype.

Several efforts have been made in order to improve immunotherapy response in this subtype. The combination of immunotherapies with different target agents or conventional chemotherapeutic strategies might represent a useful and practical means to stimulate immune cell infiltration and elicit immune response (NCT02873195, NCT02291289, NCT02876224, NCT03470350).

Moreover, it has been observed that MEK inhibition induced the accumulation of T cells within the tumor cells and the major histocompatibility complex (MHC) class I upregulation in mouse models, and synergized with immune checkpoints, promoting a sustainable tumor regression [40]. These results led MEK inhibitors to have a predominant role in combination with immune based therapies in not responsive CRC subgroup. In fact, the preliminary data of the clinical trial that evaluated the combination of anti-PD-L1 and MEK inhibitor, showed promising results with efficacy of this combination also in microsatellite stable (MSS) non-hypermutated CRC patients [41]. Based on the results of this phase Ib trial, 363 chemorefractory patients have been enrolled in the IMblaze370 (COTEZO) phase III randomized clinical trial. Patients were randomized to receive atezolizumab plus cobimetinib, atezolizumab alone or regorafenib as standard therapy in the control arm [42]. The population enrolled has been selected to be MSI low or MSS in order to potentiate the effect of immunotherapies in this not immune responsive subgroup [42]. This randomized phase III clinical trial failed to meet his primary endpoint overall survival (OS). Median OS was 8.87 months (95% CI 7.00–10.61) with atezolizumab plus cobimetinib, 7.10 months (6.05–10.05) with atezolizumab, and 8.51 months (6.41–10.71) with regorafenib [42]. The failure of this clinical trial could be explained in not correct selection of enrolled patients [42]. The combination treatment of a MEK inhibitor and anti-PD-L1 might not be able to overcome the immune resistance in this non-inflamed MSS subtype of mCRC patients. The combination with additional target agent could be helpful in overcoming this immune resistance, possibly due to activation of alterative pathway.

In this scenario another strategy under investigation is the combination of immune modulators and anti-EGFR therapy in a *RAS* WT population, elucidating the notion that the immune system substantially contributes to the therapeutic effects of monoclonal antibodies (moAbs) [43], NCT02713373, NCT03442569).

The overall compendium of molecular mechanisms driving acquired resistance to anti-EGFR therapies is likely complex and biochemically converge to activate the EGFR-RAS-MAPK pathway [3, 10, 11, 18–20]. In our recent work, we have demonstrated that MEK is key downstream effectors of EGFR pathway that must be inhibit to prevent and or delay the onset of acquired resistance to anti-EGFR treatment [11]. Nevertheless, we have found that some tumors after an initial benefit to MEKi treatment, started to regrowth limiting its use [11]. In particular, in order to understand the mechanism underlying MEKi resistance, SW48-MR, HCT116-MR and LIM1215-MR resistant models have been established in our laboratory. Microarray analysis showed that several genes involved in the PD-L1 pathway were up-regulated in SW48-MR versus SW48. Moreover, genes overexpressed in MEKi-resistant cells were related to the gene signature corresponding to CMS4 subtype of CRC. To better define these resistant models, we performed a Western Blot assay and we showed that all pathways that are up regulated in CMS4 were up regulated in this resistant cell line also at protein level. In particular, moving forward the development of MEKi resistance we assisted to a shift from epithelial to mesenchymal feature with an up-regulation of PD-L1 expression and EGFR activation. These features are present in all resistant models we have generated (WT and mutated in *RAS*), underling that this mechanism is correlated to MEKi resistance independently to cell lines mutational status. The regulation of PD-L1 expression is complex and is regulated by multiple signaling pathways, including JAK/STAT [21, 22]. *STAT3* gene is also up-regulated in MEKi-resistant cells. Inhibition of STAT3 expression by siRNA reduced PD-L1 expression and restored MEKi sensitivity. Moreover, a direct correlation has been demonstrated between EGFR and PD-L1 since EGFR could have also a role in STAT3 regulation and consequently in PD-L1 regulation [23]. Exogenous activation of EGFR by TGFα stimulation in MEKi sensitive HCT116 cell lines induced PD-L1 overexpression and resistance to MEKi.

Once demonstrated the EGFR role in modulation of PD-L1 expression and subsequently development of acquired resistance to MEKi, we further evaluated the potential mechanisms responsible for EGFR activation by over-expression of EGFR ligands in this resistant model. Moreover, an up-regulation of PD-L1 expression is also more evident in the nucleus of HCT116-MR [22, 24, 25], underling also the anti-apototic capacity of this resistant cell line. Therefore, we performed an in vivo study to validate these in vitro results. MC38 and CT26 MEKi resistant colon cancer cells have been injected subcutaneously in C57BL/6 and BALB/c mice respectively. Mice have been treated with MEKi, murine EGFR inhibitor, murine PD-L1 inhibitor and their combination. The triplet combined treatment determined an almost complete suppression of tumor growth in MC38 and CT26 MEKi resistant tumors with no evidence of tumors in 4 and 3 out of 10 mice, respectively.

Several groups have already reported that EGFR mutational status has been correlated to PD-L1 expression [43–45, 46]. In particular, Chen et al in their work demonstrated that EGFR activation by EGF stimulation, exon-19 deletions, and L858R mutation could induce PD-L1 expression through p-ERK1/2/p-c-Jun [46].

## Conclusion

In conclusion with this work we propose that anti-PD-L1antibodies may be a promising treatment option for particular subtype of CRC patients. In particular the combined treatment of MEKi, EGFRi and PD-L1i could be a strategy to convert “CSM4 like” tumors towards a “CMS1 like” immune phenotype. More evidence is needed to explore the feasibility of this combination therapy before this strategy could be translated into clinical practice. Understanding the molecular basis of interaction between cytotoxic chemotherapies or molecular target therapies and the immune system represents a target to be pursued in the definition of new therapeutic scenarios to improve the prognosis of patients with CRC and to better understand the molecular bases of immunomodulation.

## Supplementary information

**Additional file 1: Figure S1. A.** Immunofluorescence analysis of the indicate antibody followed by secondary antibody labeled with Alexa Fluor 488 in the HCT116 and HCT116 MR cells. DAPI was included to stain the nucleus. **B.** PD-L1 has a protective role in the nucleus. Western blot analysis was performed to evaluate Caspase 9, Caspase 3 and Bcl2 expression in HCT116 and HCT116 MR cells. **C.** Sensitivity of LIM1215 and LIM1215 MR cells to the increasing concentrations of BAY86–9766 (0.01–10 μM) after 96 h treatment and evaluated for proliferation by MTT staining. The results are the average ± SD of three independent experiments each done in triplicate. **D.** Analysis of intracellular signaling pathways by Western blot analysis in LIM1215 and LIM1215-MR cells. Total cell protein extracts (50 μg) were subjected to immunoblotting with the indicated antibodies, as described in Materials and Methods. Anti-tubulin antibody was used for normalization of protein extract content.

**Additional file 2: Figure S2. A.** Mice bearing MC38 and CT26 cells were treated continuously by oral gavage injection with vehicle or MEK inhibitor (BAY86–9766) (25 mg/kg every day for 5 days a week) (*n* = 8 per group). Treatments started when tumours reached volumes of 200–300 mm^3^. Animals were sacrificed when tumours reached 2.000 mm^3^ in size. Tumours from the MEK inhibitor-treated group, were removed, digested and suspended as a single cell, which were propagated in in vitro culture. **B**. Sensitivity of MC38, MC38-MR, CT26 and CT26-MR cells to the increasing concentrations of BAY86–9766 (0.01–10 μM) after 96 h treatment and evaluated for proliferation by MTT staining. The results are the average ± SD of three independent experiments each done in triplicate.

## Data Availability

The datasets generated during and/or analyzed during the current study are available from the corresponding author on reasonable request.

## Abbreviations

BrdU: 5-bromo-2-deoxyuridine
CCL2: C-C motif chemokine ligand 2
CMS: Consensus Molecular Subtypes
CO_2_: Carbon dioxide
CRC: Colorectal Cancer
CRCSC: Colorectal Cancer Subtyping Consortium
CXCL12: Chemokine (C-X-C motif)12
DAPI: 4′,6-Diamidino-2-phenylindole dihydrochloride
DMSO: dimethylsulfoxide
EGFR: Epidermal Growth Factor Receptor
EGFRi: EGFR inhibitor
EMILIN2: Elastin microbril interfacer 2
EMT: Epithelial–mesenchymal transition
FBS: Fetal Bovine Serum
FC: Fold-change
FDR: False Discovery Rate
FGF13: Fibroblast Growth Factor 13
FGF3: Fibroblast Growth Factor 3
GSEA: Gene Set Enrichment Analyses
ICAM4: Intercellular adhesion molecule 4
IGFBP3: Insulin-like growth factor binding protein 3
IGFBP7: Insulin-like growth factor binding protein 7
IPA: Ingenuity Pathways Analysis
JAK/STAT: Janus kinase signal transducer and activation of transcription
JAK3: Janus kinase 3
mAbs: Monoclonal antibodies
mCRC: Metastatic Colorectal Cancer
MDSCs: Myeloid-derived suppressor cells
MEKi: MEK inhibitor
MHC: Major histocompatibility complex
MMP1: Matrix MetalloPeptidase 1
MMP19: Matrix MetalloPeptidase 19
MMP28: Matrix MetalloPeptidase 28
MMP7: Matrix MetalloPeptidase 7
MMP9: Matrix MetalloPeptidase 9
MSI: Microsatellite instability
MSI-H: Microsatellite instability-high
MSS: Microsatellite stable
OS: Overall Survival
PBS: Phosphate Buffered Saline
PDGFRB: Platelet-derived Growth Factor Receptor, beta polypeptide
PD-L1i: PD-L1inhibitor
s.c.: Subcutaneously
SDS: Sodium Dodecyl Sulphate
SLUG: SNAIL family zinc Finger 2
SMAD4: SMAD family member
SMURF: SMAD specific E3 ubiquitin protein ligase
SNAI1: SNAIL family zinc Finger 1
SNAI3: SNAIL family zinc Finger 3
STAT1: Signal Transducer and Activator of Trascription 1
STAT2: Signal Transducer and Activator of Trascription 2
STAT3: Signal Transducer and Activator of Trascription 3
STAT4: Signal Transducer and Activator of Trascription 4
TAB2: Transforming Growth Factor beta activated kinase 2
TAB3: Transforming Growth Factor beta kinase 3
TGFA: Transforming Growth Factor alpha
TGFB1: Transforming Growth Factor beta 1
TGFβ: Transforming Growth Factor Receptor beta
TME: Tumor microenvironment
TWIST1: Twist homolog 1 (Drosophila)
VIM: Vimentin
WT: Wild Type
