# Supplementary figures and images for: Triple blockade of EGFR, MEK and PD-L1 has antitumor activity in colorectal cancer models with constitutive activation of MAPK signaling and PD-L1 overexpression
Source: J Exp Clin Cancer Res. 2019 Dec 16;38:492. doi: 10.1186/s13046-019-1497-0 (PMC6915948; doi:10.1186/s13046-019-1497-0)

Supplementary Figure 1

**A**

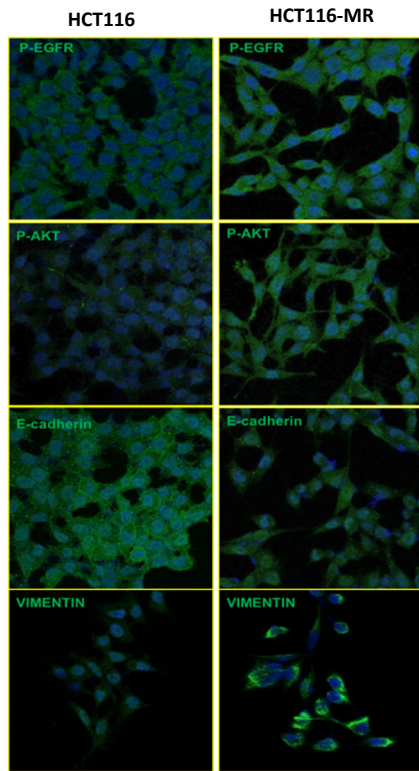

**B**

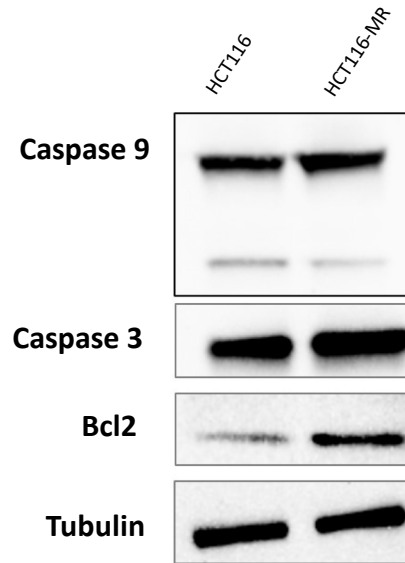

**C**

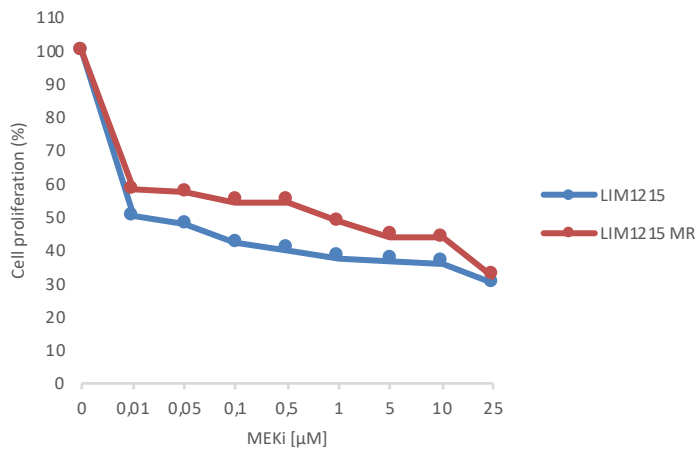

**D**

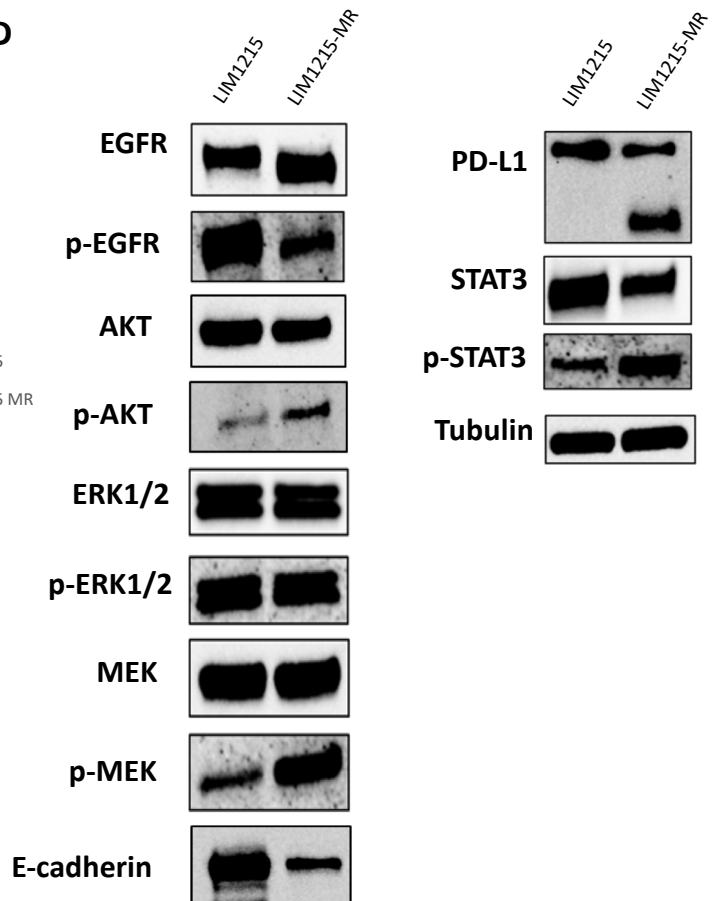

|            | IC <sub>50</sub> MEKi [μM] |
|------------|----------------------------|
| LIM1215    | 0.01                       |
| LIM1215-MR | 1                          |

Supplement: Supplementary file 1 — Additional file 1: Figure S1. A. Immunofluorescence analysis of the indicate antibody followed by secondary antibody labeled with Alexa Fluor 488 in the HCT116 and HCT116 MR cells. DAPI was included to stain the nucleus. B. PD-L1 has a protective role in the nucleus. Western blot analysis was performed to evaluate Caspase 9, Caspase 3 and Bcl2 expression in HCT116 and HCT116 MR cells. C. Sensitivity of LIM1215 and LIM1215 MR cells to the increasing concentrations of BAY86–9766 (0.01–10 μM) after 96 h treatment and evaluated for proliferation by MTT staining. The results are the average ± SD of three independent experiments each done in triplicate. D. Analysis of intracellular signaling pathways by Western blot analysis in LIM1215 and LIM1215-MR cells. Total cell protein extracts (50 μg) were subjected to immunoblotting with the indicated antibodies, as described in Materials and Methods. Anti-tubulin antibody was used for normalization of protein extract content. [file 13046_2019_1497_MOESM1_ESM.pdf]

Supplementary Figure 2

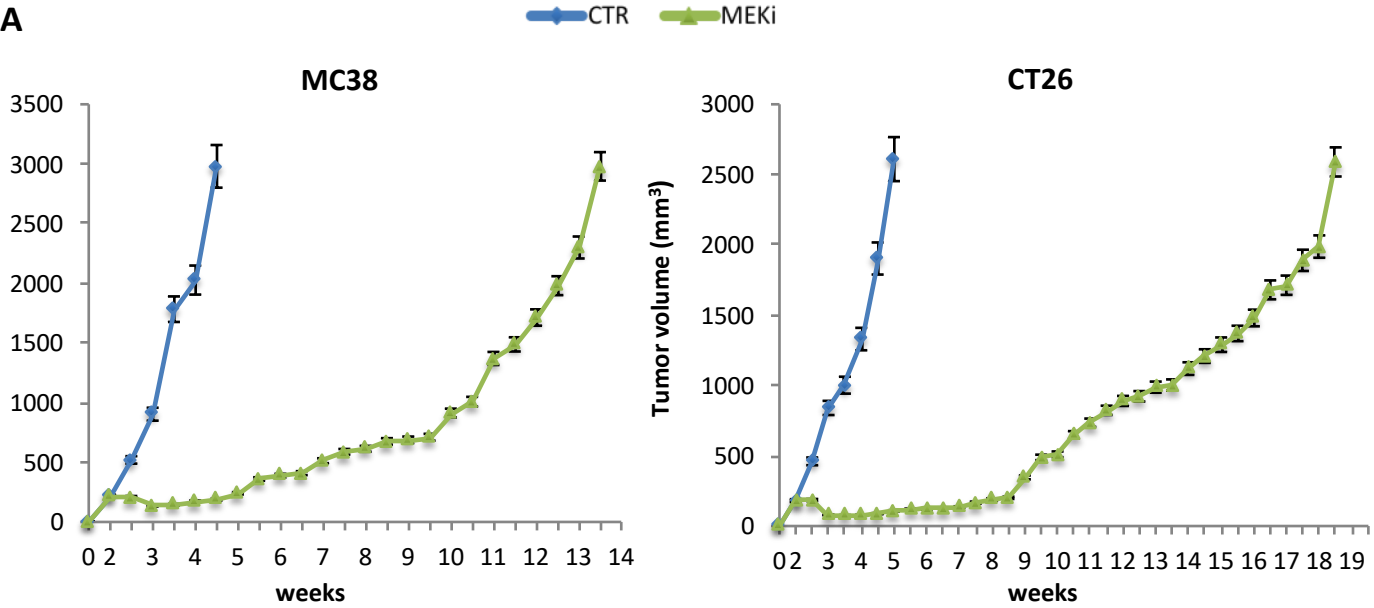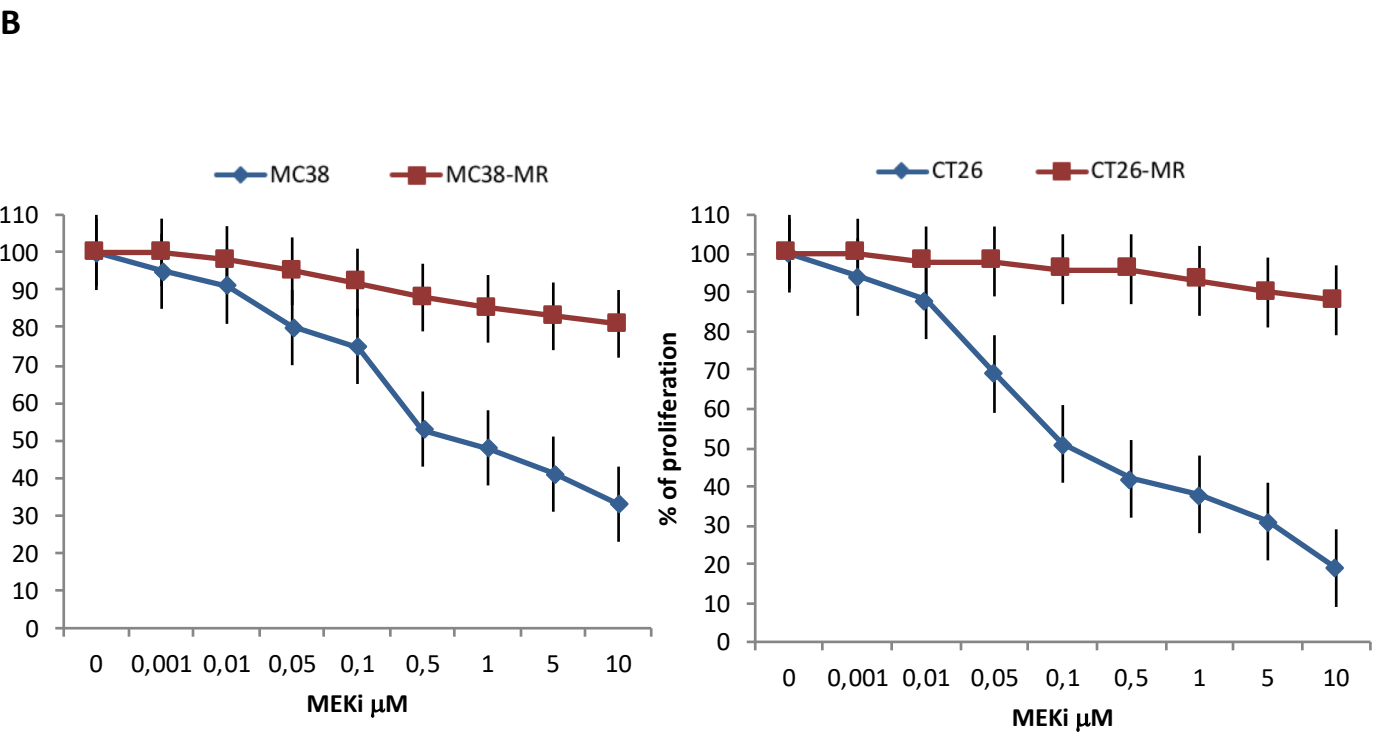

|         | IC <sub>50</sub> MEKi [ $\mu$ M] |
|---------|----------------------------------|
| MC38    | 0.5                              |
| MC38-MR | >10                              |
| CT26    | 0.1                              |
| CT26-MR | >10                              |

Supplement: Supplementary file 2 — Additional file 2: Figure S2. A. Mice bearing MC38 and CT26 cells were treated continuously by oral gavage injection with vehicle or MEK inhibitor (BAY86–9766) (25 mg/kg every day for 5 days a week) (n = 8 per group). Treatments started when tumours reached volumes of 200–300 mm3. Animals were sacrificed when tumours reached 2.000 mm3 in size. Tumours from the MEK inhibitor-treated group, were removed, digested and suspended as a single cell, which were propagated in in vitro culture. B. Sensitivity of MC38, MC38-MR, CT26 and CT26-MR cells to the increasing concentrations of BAY86–9766 (0.01–10 μM) after 96 h treatment and evaluated for proliferation by MTT staining. The results are the average ± SD of three independent experiments each done in triplicate. [file 13046_2019_1497_MOESM2_ESM.pdf]
